# Oocytes/Embryos Banking: A Vague Hope for Poor Responder Women

**Published:** 2018

**Authors:** Mohammad Reza Sadeghi

One of the main challenges in management of female factor infertility is poor responders and low response to stimulation in aged and even younger women. Usually, it is not infrequent and varies between 9 to 24% in different reports (1). This wide range of incidence is due to the absence of a obvious report in literature. But in most articles, it is defined as suboptimal response of stimulation protocols for recruitment of sufficient numbers of follicles which are associated with diminution of retrieved oocytes, cycle cancellation and significantly lower pregnancy rate. In spite of difficulty in prediction, awareness of its occurrence is extremely important for individualization of treatment protocol according to each patient’s condition (2). There are different sterategies in the managment of poor responder patients to obtain a maximum number of oocyte with best quality. Different stimulation protocols for poor responder women using wide doses and types of gonadotropins have been recommended over the past three decads; in fact, none of them have been successful and hence, the old question still remains which investigates the best strategy for management of poor responders. These protocoles include high doses of gonadotropins against the standard dose for normal responders, short and ultrashort, mini- and microdose flareup regimen of GnRH agonists, and GnRH anntagonists. In addition, further approches have been applied to potentiate the effect of the above mentioned protcoles like administration of estradiol in luteal phase of cycle, administration of recombinant LH during gonadotropin stimulation, administration of growth hormone (GH), oral administration of dehydroepiandrosterone (DHEA) as an androgen, improvement of ovarian blood flow and vascularity through administration of asprin, natural IVF cycle as a simple and inexpensive protocol, and also oocyte and embryo banking (1). There are many contradictory results on advantages or rejection/acceptance and even the null effect of these protocols; some of which are overruled and some others are still commonly used to manage these patients. Introduction of vitrification technique to IVF clinics during the past decade revolutionizes all aspects of reproductive sciences especially fertility preservation for women at risk of diminishing their ovarian reserve over the time. Accumulation of oocytes or embryos from consecutive stimulation cycles is currently applied to increase the success rate of poor responders. Theoretically, accumulation of oocytes/embryos improves the chances of pregnancy by simulation of poor responders with normo-responder women status (3). However, this hypothesis may increase the rate of patients drop-out for continuing successive cycles and also deleterious effects of vitrification–warming on supernumerary oocytes/embryos. The vitrification-thawing procedure for oocyte/embryo is highly effective, safe and easy to apply. The results are highly related to expertise of embryologist so that in well designed laboratory with expert embryologist, survival rate is near to 100% and fertilization, cleavage and blastocyst rates of the thawed oocytes are similar to fresh ones. Therefore, the effect of freezing-thawing on oocytes and especially embryos is not significant and negligible. Other reasons to use this procdure for poor responders is increasing the chances of pregnancy, reducing the costs and also reducing dropout of patients for continuing treatment (4). Since the chance of fertilization and development of each oocyte is independent of other, when the oocytes do not have the necessary potential to develop to blastocyst stage, increasing their number during successive cycles would not affect the success rate, especially regarding the fact that most IVF clinics perform elective single embryo transfer. Perhaps the only oocyte banking advantage is reduction in the cost of fertilization and embryo transfer for multiple cycles, although the cost of oocyte freezing in successive cycles and ultimately, their thawing compensate this cost reduction. The other advantage of oocytes/embryos banking in poor responders is for pre-implantation genetics screening (PGS) cycles. It is confirmed that the increase of aneuploidy rate in oocytes and resulting embryos is correlated with aging in women (5). However, the application of PGS to improve pregnancy rate in late reproduction age and also youger couples is uncertain and questionable. The focus of IVF clinics on PGS has waxed and waned during the last dacade due to its technical insufficiency, but it has risen again following introduction of next generation sequencing (NGS) technology and trophectoderm biopsy of blastocyst at the begining of current decade. However, increasing the number of embryos in techniques such as FISH and CGH array will reduce the cost of screening and increase the chance to find an euploid embryo, but at recent, the cost of NGS of each embryo is independent and finding euploid embryo among low number of embryos at one cycle will reduce the cost of further embryo screening and further IVF cycles. Regarding oocytes/embryos banking to avoid poor responders dropout, it should be noted that the practice seems to be so selfish and a type of distraint in IVF clinics for future referral of patients. Whenever a physician explains clearly the treatment process and chance of success at any stage, the patient will surely trust his physician and will accompany as long as the doctor advises them to continue further cycles. Overall, the hopeful result of an IVF cycle is at least two embryos with at least 8 cells at cleavage stage or one top quality blastocyst, so we must understand this concept of success at each cycle before starting another cycle. Currently, oocytes/embryos banking is advised and used in poor responder cycles as an effective procedure to increase pregnancy rate, but there is limited evidence of the triumph of this method in poor responders, and even in some cases, its effectiveness has been questioned. Therefore, prescribing oocytes/embryos banking to couples requires further studies and providing strong evidence for increasing pregnancy rate following consecutive cycles and oocytes/embryos storage.
